# Activation of the intermediate sum in intentional and automatic calculations

**DOI:** 10.3389/fpsyg.2015.01512

**Published:** 2015-10-02

**Authors:** Yael Abramovich, Liat Goldfarb

**Affiliations:** The Attention Laboratory, Edmond J. Safra Brain Research Center for the Study of Learning Disabilities, Department of Learning Disabilities, University of HaifaHaifa, Israel

**Keywords:** arithmetic problems, numerical cognition, inhibition, automaticity and control, addition problems, intermediate sum

## Abstract

Most research investigating how the cognitive system deals with arithmetic has focused on the processing of two addends. Arithmetic that involves more addends has specific cognitive demands such as the need to compute and hold the intermediate sum. This study examines the intermediate sums activations in intentional and automatic calculations. Experiment 1 included addition problems containing three operands. Participants were asked to calculate the sum and to remember the digits that appeared in the problem. The results revealed an interference effect in which it was hard to identify that the digit representing the intermediate sum was not actually one of the operands. Experiment 2, further examined if the intermediate sum is activated automatically when a task does not require calculation. Here, participants were presented with a prime of an addition problem followed by a target number. The task was to determine whether the target number is odd or even, while ignoring the addition problem in the prime. The results suggested that the intermediate sum of the addition problem in the prime was activated automatically and facilitated the target. Overall, the implications of those findings in the context of theories that relate to cognitive mathematical calculation is further discussed.

## Introduction

Arithmetic is a branch of mathematics that deals with numbers and their addition, subtraction, multiplication, and division. Children learn arithmetic as part of the school curriculum and both children and adults encounter and process simple arithmetic tasks, such as calculating change, in everyday life activities. Arithmetic is such a basic operation that there is evidence that even infants can solve simple arithmetic problems (Wynn, 1992; McCrink and Wynn, 2004).

Simple arithmetic processing can occur automatically, without a specific instruction to perform the task. For example, Lefevre et al. (1988), LeFevre and Kulak (1994) investigated the automaticity of arithmetic processing. In their experiment an arithmetic problem containing a pair of digits (e.g., 4 + 3) was presented, followed by a target digit. The participants’ task was to determine whether the target digit was one of the digits that appeared in the initial pair (they were not requested to solve the arithmetic problem). When the target digit was the sum of the preceding pair of digits (e.g., the target 7 preceded by the pair 4 + 3) rejection times were longer than when the target digit was not the sum of the preceding pair of digits (e.g., the target 9 preceded by the pair 4 + 3). This difference in response time (RT) between the two conditions is the interference effect, and noticeably, it occurred automatically, where there was no need to solve the arithmetic problem (participants simply had to remember and then match numeric symbols). A similar interference effect was also found with multiplication facts (Thibodeau et al., 1996).

In another study, Jackson and Coney (2005, 2007) found evidence of facilitation and inhibition effects in a numerical priming task. In that study a target number appeared on the screen (e.g., 14) and participants were asked to name it. Each target was preceded by a prime that was composed of an arithmetic problem. The sum of the problem was either congruent to the target (e.g., 6 + 8), incongruent (e.g., 2 + 3), or neutral (e.g., X + Y or 0 + 0). RT in the congruent condition was faster than in the neural condition and RT in the incongruent condition was slower than in the neutral condition.

A recent study that investigated unconscious human semantic processing boundaries, found that humans can also unconsciously solve complex arithmetic equations that require multiple steps (e.g., 9–3–4; Sklar et al., 2012).

For the last couple of decades, neuro-cognition researchers have been trying to investigate how the cognitive system deals with arithmetic and how arithmetic knowledge is organized and accessed in the brain. Most models describe the solving of simple arithmetic problems by skilled adults as a process of fact retrieval from memory (Ashcraft, 1982; Dehaene and Cohen, 1995). For example, in Ashcraft’s associative network retrieval model arithmetic facts are stored in a network of stored associations. The arithmetic problem addends and their matching result are presented as nodes. Retrieval of arithmetic facts (e.g., 2 + 5 = 7) occurs by spreading activation from the number nodes that appear in the problem (e.g., 2 and 5) through associative links to related number nodes such as the sum (e.g., 7). It is assumed that the process of spreading activation from the presented nodes to the related nodes is automatic.

Not all arithmetic involves direct accessing of arithmetic facts in long term memory, especially when the arithmetic problem is less familiar or when it is a complex one. Examples of these kinds of calculations are the multi addends arithmetic task (e.g., 3 + 4 + 7) and the multi-digit arithmetic task (e.g., 16 + 26), which are performed via a procedural process in which the calculation is performed while holding, changing, and manipulating information in the mind. It has been suggested that in these kinds of calculations the intermediate sum might be temporarily stored in the working memory (DeStefano and LeFevre, 2004). Research investigating the procedural process of composite arithmetic tasks that involve two serial operations which share an intermediate result (that should be passed from the first operation to the second), found that participants are unable to maintain the serial flow of operations. That is, the second operation starts ahead of time and is executed partially parallel to the first operation, with the task stimulus instead of the intermediate result as input (Sackur and Dehaene, 2009).

However, most research investigating how the cognitive system deals with arithmetic has focused on the processing of two addends (e.g., 3 + 2). Little is known about the exact nature of calculation in arithmetic problems with three addends (e.g., 3 + 2 + 7) and how the cognitive system handles the intermediate sum in those calculations. The current study aims to shed a light on some aspects of this calculation. The current study consists of two experiments; the first experiment examines activation of the intermediate sum in intentional serial calculations and the second experiment examines it in automatic calculations.

## Experiment 1

Experiment 1 investigates the activation of the intermediate sum when participants perform intentional serial calculations of addition problems with three addends. When performing an intentional three addend arithmetic task, it is reasonable to assume that the intermediate sum might be activated. For example, when calculating the serial addition problem with three addends: 3 + 2 + 7, the intermediate sum 5 might be activated. If this temporary sum is indeed activated, what is the nature of this activation? Does this serial calculation also involve serial inhibitions? In other words, when one sum is calculated (e.g., the total 12) does it overwrite and inhibit the existing irrelevant intermediate sums (e.g., 5)? On the other hand, it is also possible that after the final sum is calculated the intermediate sum remains activated (although at this stage it is irrelevant and has the potential to interfere with other processes). If the intermediate sum remains active, is it activated in a way that will actually cause a confusion between it and the real problem addends (e.g., 3, 2, 7)? The current experiment will explore these questions.

**FIGURE 1:**
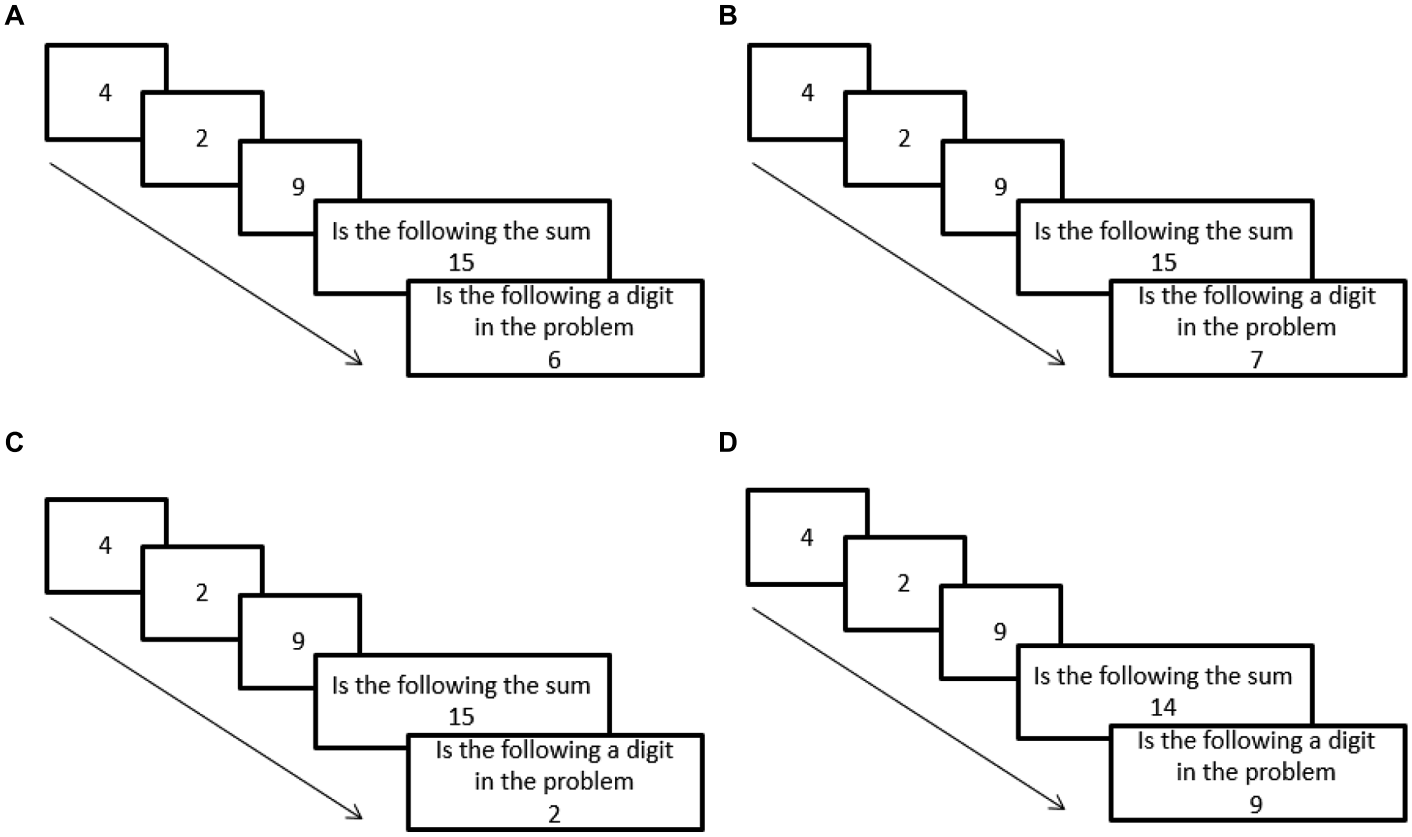
**A schematic representation of the experimental paradigm along with the various conditions are displayed.** Three addends are presented one after the other and are followed by two tasks. The first task is to identify whether a specified number is the sum of the addends. The second task is to identify whether a specified digit is one of the addends of the problem. **(A)** Condition 1 (the absent digit is the intermediate sum). Task a: correct response: “yes,” Task b: correct response: “no.” Note that in this condition the intermediate sum (6) is the digit that one needs to identity as NOT one of the addends. **(B)** Condition 2 (the absent digit is a neutral digit). Task a: correct response: “yes,” Task b: correct response: “no.”. Note that in this condition a neutral digit (7) is the digit that one needs to identity as not one of the addends. **(C)** A filler condition. Task a: correct response: “yes,” Task b: correct response: “yes.” **(D)** A filler condition. Task a: correct response: “no,” Task b: correct response: “yes.”

In this experiment participants were presented with three addends (e.g., 4, 2, 9). Then they were asked to perform two tasks: (a) calculate the sum of these addends (e.g., identify that the sum is 15) and (b) identify whether a certain digit was one of the addends in the problem displayed on the screen (e.g., identify that only the digits 4, 2, and 9 appeared on the screen as addends of the problem). For correct sum calculations (task a), RT and error rate for detecting that certain digits were not displayed (task b) were measured in two conditions of interest. In the first condition the absent digit was the intermediate sum (e.g., participants were supposed to detect that 6, which is the intermediate sum of 4 and 2, was not an addend in the addition problem). In the second condition, the absent digit was a neutral digit (e.g., participants were supposed to detect that 7 was not an addend in the addition problem; see **Figure 1**).

If the irrelevant intermediate sum is still activated after completing the addition problem, and if the activation is in a similar form to the other real problem addends, then interference by the intermediate sum digit might be observed. Namely, when participants will need to decide that a target digit did not appear on the screen, RT and/or error rate might increase when the target digit is the intermediate sum in comparison to some other neutral digits.

### Materials and Methods

The study was approved by the ethical committee of University of Haifa, Israel and was conformed to those standards. Written informed consent was obtained from all participants.

#### Participants

Nineteen adults, all females, with no diagnosed learning disabilities or attention deficits participated in the experiment. One participant was excluded due to low performance rate (she had only 53.13% correct trials). The age of the remaining 18 participants was between 23 and 33 (*M* = 27.34, *SD* = 2.87).

In return for participating, participants received course credit points, or a payment of NIS 20 (around $5).

#### Material

The stimuli displays consisted of a sequence of three digits which created an addition calculation problem set. In total there were 32 addition problem sets and each appeared twice in the experiment (see Appendix A). The addends in the addition problems were created such that each addend had a value of between two and nine, the intermediate sum was smaller than or equal to eight, and the total sum did not exceeded fifteen. In addition, the same digit didn’t appear more than once in the same problem and the intermediate sum didn’t share the same digit with either the addends or the total sum (e.g., problem set 2 + 4 + 6 with intermediate sum of 6 was excluded because it shares the same digit 6 with one of the addends). The intermediate sum differed from the addends and total sum by more than one and the order of the addends was balanced such that in half the problems the smaller of the first two addends appeared first and for the other half the larger of the first two addends appeared first.

The number presented for the sum question task could be either the correct sum (75%) or the correct sum ±1 (25%). The digit presented for the addends identifying task could be the intermediate sum (33.3%), the intermediate sum ±1 (33.3%, divided equally between plus and minus 1), or one of the addends (33.3%). All stimuli appeared in black in the center of a white screen. The digits and letters in the display had an approximate size of 0.5 cm × 0.5 cm.

#### Procedure

The experiment was constructed on the E-Prime 2.0 program. An HP Compaq computer with an Intel core i7-2600 central processor was used for presentation of stimuli and collection of data. Stimuli were presented on a Samsung 22 inch monitor, while participants sat about 60 cm from the screen. A keyboard on which the participants pressed their answer was placed on a table next to the screen. Each participant was tested individually and the experiment took about 10–15 min in total.

The participants were instructed to perform the following task: (a) Identify whether a given number is the sum of the three addends; (b) Identify whether a specified digit was one of the addends. The participants were asked to respond as quickly and accurately as possible. To ensure that participants performed the complex addition task, they were told that for identifying the sum question correctly they would earn two points and for identifying the appearance of a digit in the problem they would earn one point. They were also told that the participant that attains the highest score would receive a monetary bonus. The monetary bonus was a payment of NIS 20 (around $5). For a response of “no,” participants pressed the letter “Q” on the keyboard with their left index finger, and for a response of “yes” they pressed the letter “P” with their right index finger.

The experiment began with a practice block that contained eight trials presented randomly. Then the experimental block began.

At the beginning of each of the trials (practice and experiment) a blank screen appeared for 500 ms. Then the digits appeared on the screen one after the other and each remained for 900 ms. The digits were separated from one another by a 200 ms blank screen. Then two questions were presented, one after the other. The questions were composed of the messages “Is the following the sum” and “Is the following a digit in the problem.” Each question remained on the screen for a maximum of 3500 ms or until the participants responded. The questions were separated from one another by a 200 ms blank screen. The order of the questions was counterbalanced, half of the participants were first asked about the appearance of a digit and the other half were first asked about the sum. RT and accuracy were measured by the computer.

### Results

For correct sum calculations (task a), mean RT and error rate for detecting the absence of a digit in the addition problem (task b) were calculated for each participant in two conditions of interest: the absent digit was the intermediate sum or other neutral digit (the intermediate sum ±1).

Trials in which the participants did not answer correctly regarding the sum of the addition problem (5.56% of the group that received the digit question first and 3.82% of the group with the sum question first) were not included in the analysis. Analysis of RT for detecting the absence of a digit in the addition problem was calculated only in trials where the participants answered the two questions correctly (86.81% of the group with the digit question first and 84.72% of the group with the sum question first).

A two-way analysis of variance was applied to the RT data with type of digit (the intermediate sum or neutral) as a within participant factor and the order of questions as a between participant factor.

**FIGURE 2:**
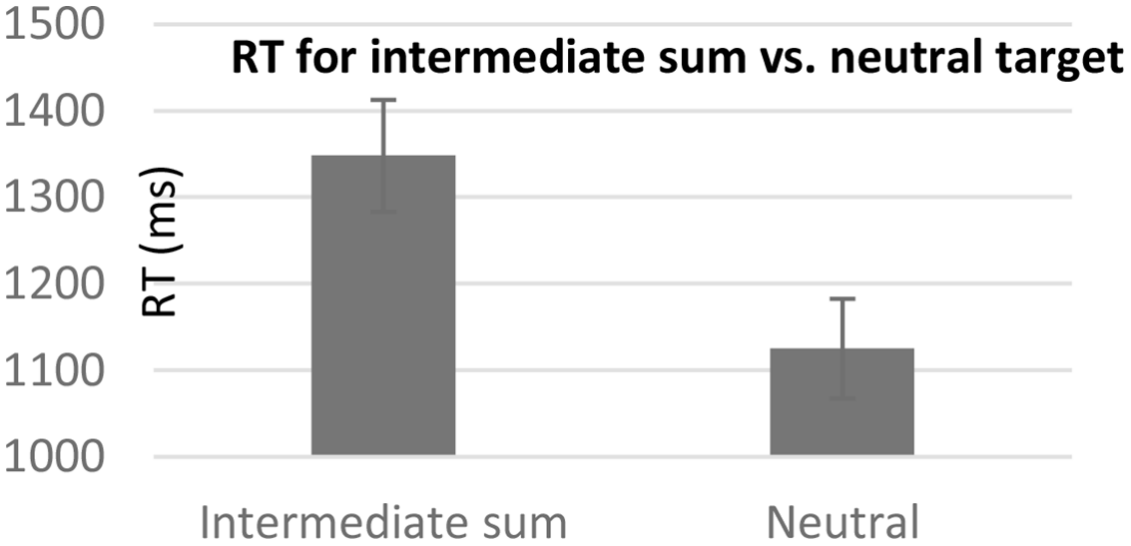
**Mean response time (RT) for rejecting a digit representing the intermediate sum versus a neutral digit.** Error bars represent standard error mean.

The results revealed that when participants had to identify that a digit was not one of the addends, RT was slower when the digit was the intermediate sum (1348 ms, *SD* = 274.76) than a neutral digit (1125 ms, *SD* = 244.54), [*F*(1,16) = 34.67, *p* < 0.001] (see **Figure 2**). This effect did not significantly interact with the order of the questions [*F*(1,16) = 2.27, *p* = 0.152].

**FIGURE 3:**
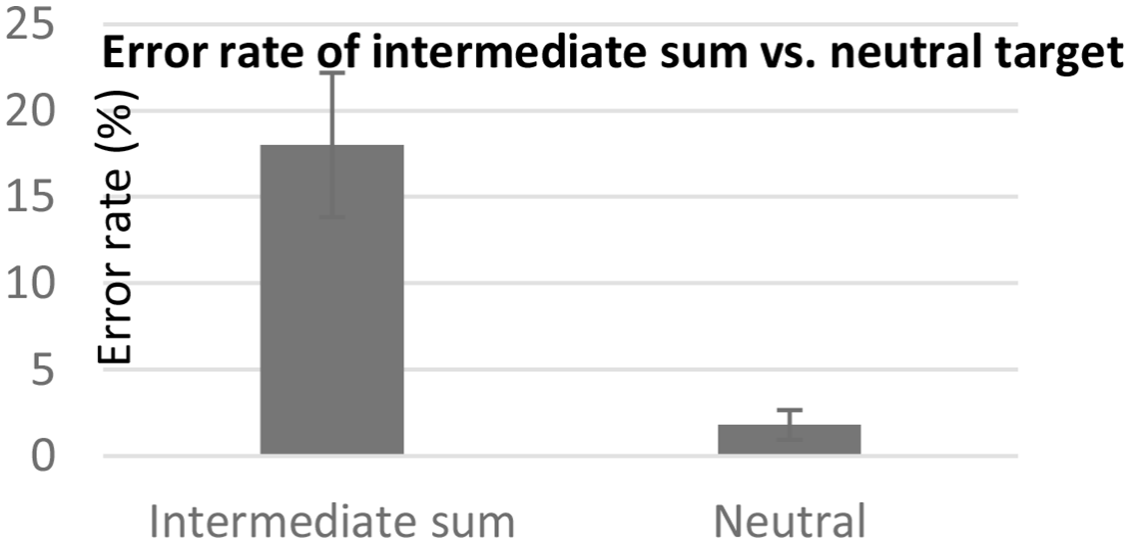
**Mean error rate for rejecting a digit representing the intermediate sum versus a neutral digit.** Error bars represent standard error mean.

Similar results were revealed in the error analysis. Participants made more mistakes in trying to identify that a digit didn’t appear in the addition problem when the digit was the intermediate sum (18.01%, *SD* = 17.75) than when it was a neutral digit (1.79%, *SD* = 3.66), [*F*(1,16) = 17.09, *p* < 0.01] (see **Figure 3**). This effect did not significantly interact with the order of the questions (*F* < 1).

Overall, the results demonstrate an interference effect that results from activation of the intermediate sum. In this task, participants were asked to decide if a certain digit was one of the addends. The results suggest that participants tend to make more mistakes and claim more times that a digit was one of the addends (when actually it was not) in the case of the intermediate sum than in the case of a neutral digit. Additionally, even for correct trials, it took participants more time to identify that the intermediate sum was not one of the addends in comparison to the time it took them to identify that a neutral digit was not one of the addends. Therefore, both the statistical analyses that compare RT and accuracy between the intermediate sum and a neutral digit suggest that there is a clear tendency to confuse between the identity of the intermediate sum and the identity of the addends, and that participants find it hard to distinguish between the intermediate sum and the real addends.

## Experiment 2

The results of the first experiment suggest that the intermediate sum remains activated even after the calculation process has been concluded. Participants find it difficult to distinguish between the intermediate sum and the addends of the addition problem. As noted, previous studies suggest that the presentation of a simple addition arithmetic problem with two addends leads to automatic activation of the total sum of the problem. This occurred although the task does not require participants to solve the addition problem, and in two addend addition problems the total sum is activated automatically even with a short exposure (Lefevre et al., 1988; LeFevre and Kulak, 1994; Jackson and Coney, 2005, 2007). Will the intermediate sum in three addend addition problems, that is, the sum of the first two addends, be activated according to the same rule? On one hand it is possible that the activation of intermediate sums is a core characteristic of the human calculating system and it is activated automatically even with a short exposure. Then again, when the task does not require calculating the exact sum of the multi addends problem, it is not clear whether a serial computation that includes activation of the intermediate sum is actually performed. There is evidence that conscious access is required for multi-step arithmetic operations in order to control the flow and passing of the information (e.g., intermediate sum) from one operation to the other (Sackur and Dehaene, 2009). Therefore, serial processes involving serial addition and transmission of the intermediate sum from one step to the other are not necessarily how the cognitive system automatically refers to multi step arithmetic problems. The cognitive system might process the stimuli by coding the overall statistics (“ensemble coding”) of the information, which might act as a parallel process. This might be similar to how the mean of objects is calculated (Chong and Treisman, 2005). In line with this possibility is evidence that ensemble statistics can be extracted from a set of abstract symbolic stimuli (e.g., arabic numbers) without awareness (Van Opstal et al., 2011). The human mind has also been shown to have the ability of fast approximate addition calculation which does not require serial processing (El Yagoubi et al., 2003), which could also be what the cognitive system does automatically when briefly presented with a multi step arithmetic task. Hence, these options will not necessarily result in calculation of the complete sum of the multi addend problem and the intermediate sum, and therefore the intermediate sum will not be activated in those cases.

**FIGURE 4:**
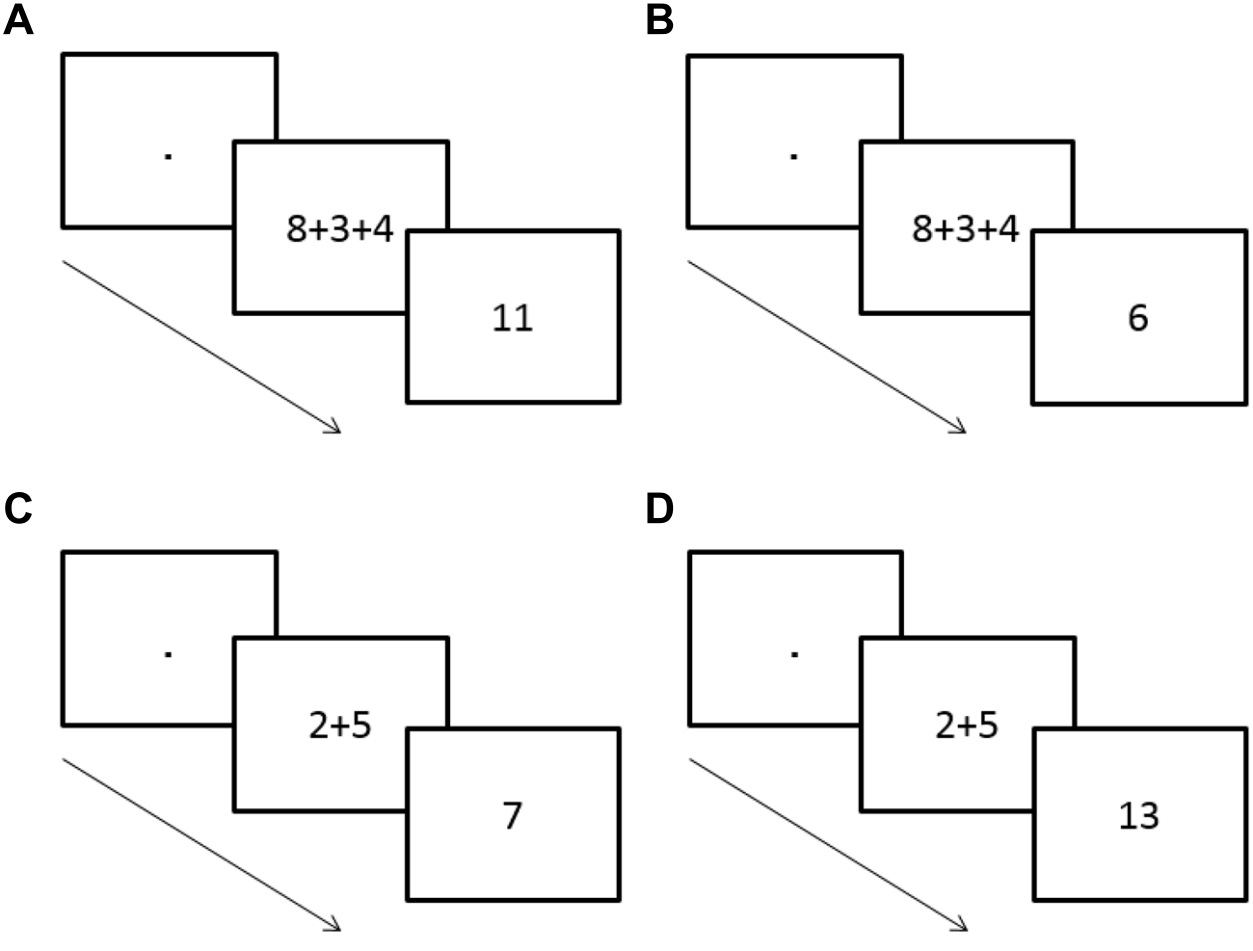
**Schematic representations of the experimental paradigm along with the various conditions are displayed.** An addition task of two or three addends is presented for 150 ms followed by a target number. The task is to determine whether the target number is odd or even, while ignoring the arithmetic addition problem. **(A)** The intermediate sum of two addends in a prime of a three addend problem is congruent with the target. Correct response: “odd” **(B)** The intermediate sum of two addends in a prime of a three addend problem is incongruent with the target. Correct response: “even.” **(C)** The sum of two addends in a prime of a two addend problem is congruent with the target. Correct response: “odd.” **(D)** The sum of two addends in a prime of a two addend problem is incongruent with the target. Correct response: “odd.”

The current experiment examined whether the activation of intermediate sums is a core characteristic of the human calculating system using a priming procedure with an addition problem as the prime and an intermediate sum or an incongruent number as the target. In the current experiment, participants were presented with a prime of an addition problem with two or three addends. Following the prime a target number was presented. The participants’ task was to determine whether the target number is odd or even, while ignoring the addition problem. For the two addend addition problem the target could be either congruent with the sum of the problem (e.g., prime: 2 + 5 and target: 7) or incongruent (e.g., prime: 2 + 5 and target: 13). In the three addend addition problem, the target could be either congruent with the intermediate sum of the problem (e.g., prime: 8 + 3 + 4 and target: 11) or incongruent (e.g., prime: 8 + 3 + 4 and target: 6) (see **Figure 4**). Note that here the intermediate sum in three addend addition problem is the sum of the first two addends. A faster RT for the intermediate sum (the congruent target) as opposed to an incongruent target indicates automatic activation of the intermediate sum even if the calculation is irrelevant to the task.

### Materials and Methods

The method of Experiment 2 was similar to Experiment 1 with the following changes. Twenty four adults including 1 male and 23 females took part in the experiment. In return for participating, participants received course credit points, or a payment of NIS 20 (around $5), but no monetary bonus for high achievements has been proposed since it was an easier task than Experiment 1. One female participant was excluded from the analysis due to computer failure during the experiment. The age of the remaining 23 participants was between 22 and 33 (*M* = 26.7, *SD* = 2.64). A prime of an arithmetic addition problem was presented, followed by a target number. The participants’ task was to determine whether the target number is odd or even while ignoring the arithmetic addition problem that appeared on the prime. They were asked to use their left index finger to press the letter “W” (for an “odd” response) and their right index finger to press the letter “O” (for an “even” response). The prime was an addition problem with two or three addends. For the addition problem with two addends the target could be either congruent with the sum of the problem (e.g., prime: 2 + 5 and target: 7; 16.66% of all trials) or incongruent (e.g., prime: 2 + 5 and target: 13; 16.66% of all trials). In the three addend addition problem the target could be either congruent with the intermediate sum of the problem (e.g., prime: 8 + 3 + 4 and target: 11; 16.66% of all trials) or incongruent (e.g., prime: 8 + 3 + 4 and target: 6; 16.66% of all trials). Another option was that of a target that is congruent with the total sum of the problem (e.g., prime: 6 + 2 + 7 and target: 15; 16.66% of all trials) or incongruent (e.g., prime: 6 + 2 + 7 and target: 11; 16.66% of all trials). The two latter acted as fillers. In total there were 204 addition problems (see Appendix B). The values of the addends in the addition problems were between two and nine, and the total sum was 16 or smaller. The following restrictions were made: the same digit didn’t appear more than once in the same problem. In addition, the intermediate sum, total sum, and addends didn’t have the same unit digit (e.g., problem set 2 + 3 + 7 with total sum of 12 was excluded because the unit digit of the total sum is identical to one of the addends). Also, the congruent and incongruent targets didn’t have the same unit digit as the addends, intermediate sum, or total sum (e.g., problem set 2 + 7 + 5 with incongruent target 4 was excluded because the unit digit of the total sum 14 is identical to the incongruent target). Another constraint on the incongruent target was that it couldn’t be the result of the first two or all three addends using a different operation (e.g., problem set 3 + 2 + 9 with incongruent target 6 was excluded because the incongruent target could be the result of multiplying the first two addends 3 × 2). To account for the split effect, which refers to a problem with an incorrect answer that is closer to the correct answer, requiring more time for rejection (see Ashcraft, 1992 for a review), incongruent targets differed from the intermediate sum and the total sum by at least three and from the addends by more than one.

Moreover, the total sum of the difference between incongruent and congruent targets (the total sum for the two addends condition and the intermediate sum for the three addends condition) was balanced at zero and the congruent and incongruent targets were balanced by range, the amount of times each number was used, and the amount of odds and evens. Finally, the order of the addends was also balanced such that in half the problems the smaller of the first two addends was placed on the left side and for the other half the larger of the first two addends was placed on the left side.

The approximate size of each digit was 0.4 cm × 0.4 cm. The experiment took about 20 min to complete. The experiment began with a practice block that consisted of eight trials presented randomly. Then an experimental block began. This block contained 204 trials presented at random order. At the beginning of each of the trials (practice and experiment) a fixation point of 600 ms appeared, followed by a 150 ms blank screen. Then the addition problem appeared on the screen and remained for 150 ms. The following was 500 ms of blank screen and then the target number appeared and remained on the screen for a maximum of 3000 ms or until the participant responded by pressing the appropriate letter key. Finally, there were 2000 ms of blank screen between trials.

### Results

Trials in which the participants did not answer the even/odd question correctly (1.42%) or trials with an RT of less than 300 ms or more than 1,500 ms (0.78%) were not included in the analysis.

For the remaining trials, for each participant in each condition of interest the mean RT was calculated. A two-way analysis of variance was applied to the mean RT data with prime-target congruency (congruent, incongruent) and sum type (overall sum of two addends, intermediate sum of two addends in a three addend problem) as a within participant factor.

**FIGURE 5:**
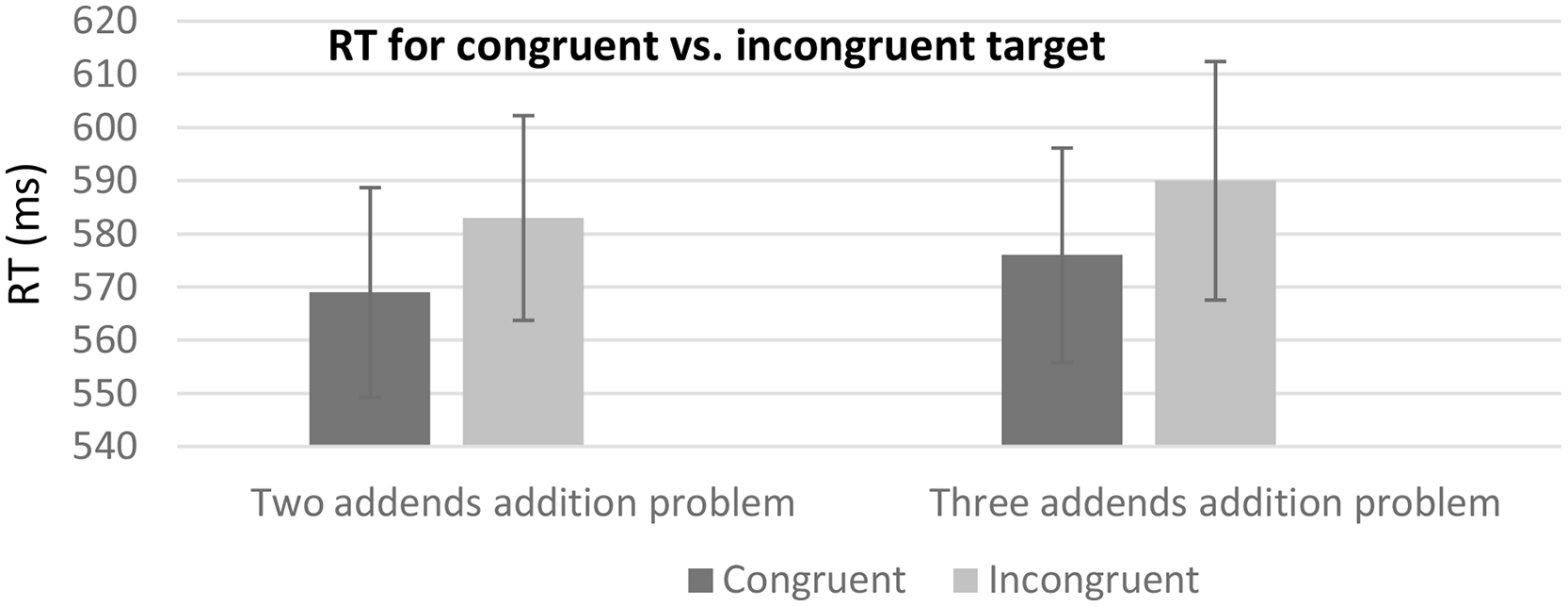
**Mean RT as a function of the different experimental conditions.** Error bars represent standard error mean.

The results for the mean RT data revealed a significant effect for the prime-target congruency condition [*F*(1,22) = 13.43, *p* < 0.01]. There was no significant main effect for the sum type condition [*F*(1,22) = 1.99, *p* = 0.17]. The interaction between the two conditions failed to reach significance (*F* < 1). Further analysis revealed that for the two addends condition it took more time to respond to the prime-target incongruent target (583 ms *SD* = 92.26) than to the prime-target congruent target (569 ms, *SD* = 94.54), *t*(22) = -2.55, *p* < 0.025. Similarly, in three addend problems, it took more time to respond when the intermediate sum of the first two addends in the prime was incongruent with the target (590 ms, *SD* = 107.52) than when it was congruent with the target (576 ms, *SD* = 96.62), *t*(22) = -2.17, *p* < 0.05, (see **Figure 5**). Due to the overall low error rate (1.42%), the error rate was not further analyzed.

Overall, the results in this experiment replicate previous research findings regarding automatic activation of the overall sum in two addend addition problems. Moreover, interestingly, the results suggest that the intermediate sum of two addends is also automatically activated with short exposure when it is part of three addend addition problems.

## General Discussion

In summary, Experiment 1 found that in an intentional serial three addend summation problem, the intermediate sum is activated. This intermediate sum representation is activated to such a degree that participants tend to confuse the intermediate sum with the actual addends in the problem. Note that the cognitive system still succeeds most of the time to distinguish between the intermediate sum and the actual addends. However, even in those cases the system still pays the price of longer RT to make this distinction compared to the neutral stimuli.

The fact that the intermediate sum remains activated even though the calculation has been completed and there is no need for the temporary intermediate sum contrasts with the calculation performed by an artificial tool such as a calculator. In a calculator for example each intermediate result runs over the preceding one and only the final result is kept and presented. In addition, Experiment 2 found that activation of the intermediate sum is a core and important feature in solving complex mathematical problems. Even when three addend addition problems are presented with short exposure and no calculation is needed, the intermediate sum of the first two addends is automatically activated (note that we do not preclude the option that it is possible that in addition the cognitive system automatically activates the sum of two other random addends that appeared in the addition problem). Also note that in automatic calculations the intermediate sum is activated even though the cognitive systems theoretically have the ability to perform parallel computations such as ensemble coding (Van Opstal et al., 2011) or approximate calculations (El Yagoubi et al., 2003) which do not require serial processing (and consequently do not require activation of the intermediate sum).

Intermediate sum activation has both advantages and disadvantages. On the one hand, multi step arithmetic that is performed serially requires activation of intermediate results. Loss of that information before the total sum is computed would result in calculation errors (Hitch, 1978). Additionally, note that the automatic intermediate sum activation found in Experiment 2 possibly enables the cognitive system to reduce the cognitive load from complex arithmetic problems and might result in faster and more efficient processing. In fact, studies investigating individual differences in arithmetic found that skilled individuals show more efficient automatic access and retrieval of arithmetic facts (LeFevre and Kulak, 1994; Jackson and Coney, 2007). On the other hand, high uncontrolled activations that result from either automatic or intentional processes might also have a negative effect as they might generate a significant load on the cognitive system when it is irrelevant. These kinds of activations have a strong potential to interfere, as observed in the current research where participants presented tendency to confuse between the input data of the arithmetic problem and the intermediate results. It is expected that an intermediate sum that is temporary and no longer relevant for further processing once the calculation has been completed, will be de-activated or at least that its activation will be to a degree that will not strongly interfere with distinguishing between the temporary calculation and the actual operands of the problem. In order to practice math, one has to be able to distinguish between addends of the problem and intermediate calculations, and inability to efficiently distinguish between them might explain some errors and difficulties. It is currently unclear how the cognitive system handles and resolves these interferences and further research of this issue is required.

Another issue that different calculation models need to address, based on the current findings, is how parallel processing can theoretically be possible. In the introduction we noted that when a task does not require calculating the exact sum of the multi addends problem, the approximate sum of the addends can potentially be estimated concurrently. The cognitive system has a remarkable ability to conduct statistical summary perception (SSP), which relates to the ability to immediately perceive summary properties (e.g., average size) from a set of objects (Ariely, 2001; Chong and Treisman, 2005; Alvarez and Oliva, 2008). Hence, theoretically it is also possible for the approximate sum of several addends to be processed simultaneously, similar to the calculation of these statistical summaries (Van Opstal et al., 2011). Although this parallel option is interesting, more development is needed to show how this option can be possible. Specifically, if as found in the current work the intermediate sum is activated automatically even when no calculation is needed, then why is this extra activation of the intermediate sum not added to the total parallel sum as well?

Finally, we would like to note that the current study describes behavioral effects in the context of the intermediate sum of three addend arithmetic problems. Although the processes involved in two addend arithmetic problems have been studied over the past years, only few studies have directly addressed the unique problems involved in more complex arithmetic problems. The lack of sufficient research in this field is in contrast to the complex arithmetic problems one experiences in daily life or at school. The more studies are conducted in this field, the better we will be able to understand the human calculation system.

## Conflict of Interest Statement

The authors declare that the research was conducted in the absence of any commercial or financial relationships that could be construed as a potential conflict of interest.
