# Supplementary material for: Activation of the intermediate sum in intentional and automatic calculations
Source: Front Psychol. 2015 Oct 2;6:1512. doi: 10.3389/fpsyg.2015.01512 (PMC4591477; doi:10.3389/fpsyg.2015.01512)
Supplement: Supplementary file 1 [file Data_Sheet_1.DOCX]

Appendix A

Experiment 1 addition problem sets.

| *Addend 1* | *Addend 2* | *Addend 3* |
| --- | --- | --- |
| 2 | 3 | 7 |
| 2 | 3 | 8 |
| 2 | 3 | 9 |
| 2 | 4 | 3 |
| 2 | 4 | 8 |
| 2 | 4 | 9 |
| 2 | 5 | 3 |
| 2 | 5 | 4 |
| 2 | 6 | 3 |
| 2 | 6 | 4 |
| 2 | 6 | 5 |
| 3 | 2 | 7 |
| 3 | 2 | 8 |
| 3 | 2 | 9 |
| 3 | 4 | 2 |
| 3 | 4 | 5 |
| 3 | 5 | 2 |
| 3 | 5 | 4 |
| 3 | 5 | 6 |
| 4 | 2 | 3 |
| 4 | 2 | 8 |
| 4 | 2 | 9 |
| 4 | 3 | 2 |
| 4 | 3 | 5 |
| 5 | 2 | 3 |
| 5 | 2 | 4 |
| 5 | 3 | 2 |
| 5 | 3 | 4 |
| 5 | 3 | 6 |
| 6 | 2 | 3 |
| 6 | 2 | 4 |
| 6 | 2 | 5 |

Appendix B

Experiment 2 addition problem sets.

| *Prime* | *Target* | *Condition* | *Congruency* |
| --- | --- | --- | --- |
| 2 + 3 | 5 | Two addends | Congruent |
| 2 + 4 | 6 | Two addends | Congruent |
| 2 + 4 | 6 | Two addends | Congruent |
| 2 + 5 | 7 | Two addends | Congruent |
| 2 + 6 | 8 | Two addends | Congruent |
| 2 + 9 | 11 | Two addends | Congruent |
| 2 + 9 | 11 | Two addends | Congruent |
| 3 + 2 | 5 | Two addends | Congruent |
| 3 + 4 | 7 | Two addends | Congruent |
| 3 + 5 | 8 | Two addends | Congruent |
| 3 + 6 | 9 | Two addends | Congruent |
| 3 + 8 | 11 | Two addends | Congruent |
| 3 + 9 | 12 | Two addends | Congruent |
| 4 + 2 | 6 | Two addends | Congruent |
| 4 + 2 | 6 | Two addends | Congruent |
| 4 + 3 | 7 | Two addends | Congruent |
| 4 + 5 | 9 | Two addends | Congruent |
| 5 + 2 | 7 | Two addends | Congruent |
| 5 + 3 | 8 | Two addends | Congruent |
| 5 + 4 | 9 | Two addends | Congruent |
| 5 + 6 | 11 | Two addends | Congruent |
| 5 + 9 | 14 | Two addends | Congruent |
| 5 + 9 | 14 | Two addends | Congruent |
| 6 + 2 | 8 | Two addends | Congruent |
| 6 + 3 | 9 | Two addends | Congruent |
| 6 + 5 | 11 | Two addends | Congruent |
| 6 + 7 | 13 | Two addends | Congruent |
| 7 + 6 | 13 | Two addends | Congruent |
| 8 + 3 | 11 | Two addends | Congruent |
| 9 + 2 | 11 | Two addends | Congruent |
| 9 + 2 | 11 | Two addends | Congruent |
| 9 + 3 | 12 | Two addends | Congruent |
| 9 + 5 | 14 | Two addends | Congruent |
| 9 + 5 | 14 | Two addends | Congruent |
| 2 + 3 | 8 | Two addends | Incongruent |
| 2 + 4 | 9 | Two addends | Incongruent |
| 2 + 4 | 11 | Two addends | Incongruent |
| 2 + 5 | 13 | Two addends | Incongruent |
| 2 + 6 | 14 | Two addends | Incongruent |
| 2 + 9 | 5 | Two addends | Incongruent |
| 2 + 9 | 6 | Two addends | Incongruent |
| 3 + 2 | 8 | Two addends | Incongruent |
| 3 + 4 | 11 | Two addends | Incongruent |
| 3 + 5 | 11 | Two addends | Incongruent |
| 3 + 6 | 14 | Two addends | Incongruent |
| 3 + 8 | 6 | Two addends | Incongruent |
| 3 + 9 | 7 | Two addends | Incongruent |
| 4 + 2 | 9 | Two addends | Incongruent |
| 4 + 2 | 11 | Two addends | Incongruent |
| 4 + 3 | 11 | Two addends | Incongruent |
| 4 + 5 | 12 | Two addends | Incongruent |
| 5 + 2 | 13 | Two addends | Incongruent |
| 5 + 3 | 11 | Two addends | Incongruent |
| 5 + 4 | 12 | Two addends | Incongruent |
| 5 + 6 | 8 | Two addends | Incongruent |
| 5 + 9 | 7 | Two addends | Incongruent |
| 5 + 9 | 11 | Two addends | Incongruent |
| 6 + 2 | 14 | Two addends | Incongruent |
| 6 + 3 | 14 | Two addends | Incongruent |
| 6 + 5 | 8 | Two addends | Incongruent |
| 6 + 7 | 9 | Two addends | Incongruent |
| 7 + 6 | 9 | Two addends | Incongruent |
| 8 + 3 | 6 | Two addends | Incongruent |
| 9 + 2 | 5 | Two addends | Incongruent |
| 9 + 2 | 6 | Two addends | Incongruent |
| 9 + 3 | 7 | Two addends | Incongruent |
| 9 + 5 | 7 | Two addends | Incongruent |
| 9 + 5 | 11 | Two addends | Incongruent |
| 2 + 3 + 6 | 5 | Three addends | Congruent |
| 2 + 4 + 7 | 6 | Three addends | Congruent |
| 2 + 4 + 9 | 6 | Three addends | Congruent |
| 2 + 5 + 9 | 7 | Three addends | Congruent |
| 2 + 6 + 3 | 8 | Three addends | Congruent |
| 2 + 9 + 3 | 11 | Three addends | Congruent |
| 2 + 9 + 4 | 11 | Three addends | Congruent |
| 3 + 2 + 6 | 5 | Three addends | Congruent |
| 3 + 4 + 8 | 7 | Three addends | Congruent |
| 3 + 5 + 6 | 8 | Three addends | Congruent |
| 3 + 6 + 2 | 9 | Three addends | Congruent |
| 3 + 8 + 4 | 11 | Three addends | Congruent |
| 3 + 9 + 4 | 12 | Three addends | Congruent |
| 4 + 2 + 7 | 6 | Three addends | Congruent |
| 4 + 2 + 9 | 6 | Three addends | Congruent |
| 4 + 3 + 8 | 7 | Three addends | Congruent |
| 4 + 5 + 7 | 9 | Three addends | Congruent |
| 5 + 2 + 9 | 7 | Three addends | Congruent |
| 5 + 3 + 6 | 8 | Three addends | Congruent |
| 5 + 4 + 7 | 9 | Three addends | Congruent |
| 5 + 6 + 2 | 11 | Three addends | Congruent |
| 5 + 9 + 2 | 14 | Three addends | Congruent |
| 5 + 9 + 2 | 14 | Three addends | Congruent |
| 6 + 2 + 3 | 8 | Three addends | Congruent |
| 6 + 3 + 2 | 9 | Three addends | Congruent |
| 6 + 5 + 2 | 11 | Three addends | Congruent |
| 6 + 7 + 2 | 13 | Three addends | Congruent |
| 7 + 6 + 2 | 13 | Three addends | Congruent |
| 8 + 3 + 4 | 11 | Three addends | Congruent |
| 9 + 2 + 3 | 11 | Three addends | Congruent |
| 9 + 2 + 4 | 11 | Three addends | Congruent |
| 9 + 3 + 4 | 12 | Three addends | Congruent |
| 9 + 5 + 2 | 14 | Three addends | Congruent |
| 9 + 5 + 2 | 14 | Three addends | Congruent |
| 2 + 3 + 6 | 8 | Three addends | Incongruent |
| 2 + 4 + 7 | 9 | Three addends | Incongruent |
| 2 + 4 + 9 | 11 | Three addends | Incongruent |
| 2 + 5 + 9 | 13 | Three addends | Incongruent |
| 2 + 6 + 3 | 14 | Three addends | Incongruent |
| 2 + 9 + 3 | 5 | Three addends | Incongruent |
| 2 + 9 + 4 | 6 | Three addends | Incongruent |
| 3 + 2 + 6 | 8 | Three addends | Incongruent |
| 3 + 4 + 8 | 11 | Three addends | Incongruent |
| 3 + 5 + 6 | 11 | Three addends | Incongruent |
| 3 + 6 + 2 | 14 | Three addends | Incongruent |
| 3 + 8 + 4 | 6 | Three addends | Incongruent |
| 3 + 9 + 4 | 7 | Three addends | Incongruent |
| 4 + 2 + 7 | 9 | Three addends | Incongruent |
| 4 + 2 + 9 | 11 | Three addends | Incongruent |
| 4 + 3 + 8 | 11 | Three addends | Incongruent |
| 4 + 5 + 7 | 12 | Three addends | Incongruent |
| 5 + 2 + 9 | 13 | Three addends | Incongruent |
| 5 + 3 + 6 | 11 | Three addends | Incongruent |
| 5 + 4 + 7 | 12 | Three addends | Incongruent |
| 5 + 6 + 2 | 8 | Three addends | Incongruent |
| 5 + 9 + 2 | 7 | Three addends | Incongruent |
| 5 + 9 + 2 | 11 | Three addends | Incongruent |
| 6 + 2 + 3 | 14 | Three addends | Incongruent |
| 6 + 3 + 2 | 14 | Three addends | Incongruent |
| 6 + 5 + 2 | 8 | Three addends | Incongruent |
| 6 + 7 + 2 | 9 | Three addends | Incongruent |
| 7 + 6 + 2 | 9 | Three addends | Incongruent |
| 8 + 3 + 4 | 6 | Three addends | Incongruent |
| 9 + 2 + 3 | 5 | Three addends | Incongruent |
| 9 + 2 + 4 | 6 | Three addends | Incongruent |
| 9 + 3 + 4 | 7 | Three addends | Incongruent |
| 9 + 5 + 2 | 7 | Three addends | Incongruent |
| 9 + 5 + 2 | 11 | Three addends | Incongruent |
| 2 + 3 + 6 | 11 | Fillers |  |
| 2 + 3 + 9 | 14 | Fillers |  |
| 2 + 4 + 7 | 13 | Fillers |  |
| 2 + 5 + 3 | 10 | Fillers |  |
| 2 + 5 + 4 | 11 | Fillers |  |
| 2 + 5 + 9 | 16 | Fillers |  |
| 2 + 6 + 3 | 11 | Fillers |  |
| 2 + 6 + 7 | 15 | Fillers |  |
| 3 + 2 + 6 | 11 | Fillers |  |
| 3 + 2 + 9 | 14 | Fillers |  |
| 3 + 4 + 2 | 9 | Fillers |  |
| 3 + 4 + 8 | 15 | Fillers |  |
| 3 + 5 + 2 | 10 | Fillers |  |
| 3 + 5 + 6 | 14 | Fillers |  |
| 3 + 6 + 2 | 11 | Fillers |  |
| 4 + 2 + 7 | 13 | Fillers |  |
| 4 + 3 + 2 | 9 | Fillers |  |
| 4 + 3 + 8 | 15 | Fillers |  |
| 4 + 5 + 3 | 12 | Fillers |  |
| 4 + 5 + 7 | 16 | Fillers |  |
| 5 + 2 + 3 | 10 | Fillers |  |
| 5 + 2 + 4 | 11 | Fillers |  |
| 5 + 2 + 9 | 16 | Fillers |  |
| 5 + 3 + 2 | 10 | Fillers |  |
| 5 + 3 + 6 | 14 | Fillers |  |
| 5 + 4 + 3 | 12 | Fillers |  |
| 5 + 4 + 7 | 16 | Fillers |  |
| 5 + 9 + 2 | 16 | Fillers |  |
| 6 + 2 + 3 | 11 | Fillers |  |
| 6 + 2 + 7 | 15 | Fillers |  |
| 6 + 3 + 2 | 11 | Fillers |  |
| 6 + 7 + 2 | 15 | Fillers |  |
| 7 + 6 + 2 | 15 | Fillers |  |
| 9 + 5 + 2 | 16 | Fillers |  |
| 2 + 3 + 6 | 14 | Fillers |  |
| 2 + 3 + 9 | 11 | Fillers |  |
| 2 + 4 + 7 | 10 | Fillers |  |
| 2 + 5 + 3 | 16 | Fillers |  |
| 2 + 5 + 4 | 16 | Fillers |  |
| 2 + 5 + 9 | 13 | Fillers |  |
| 2 + 6 + 3 | 15 | Fillers |  |
| 2 + 6 + 7 | 11 | Fillers |  |
| 3 + 2 + 6 | 14 | Fillers |  |
| 3 + 2 + 9 | 11 | Fillers |  |
| 3 + 4 + 2 | 15 | Fillers |  |
| 3 + 4 + 8 | 10 | Fillers |  |
| 3 + 5 + 2 | 14 | Fillers |  |
| 3 + 5 + 6 | 11 | Fillers |  |
| 3 + 6 + 2 | 15 | Fillers |  |
| 4 + 2 + 7 | 10 | Fillers |  |
| 4 + 3 + 2 | 15 | Fillers |  |
| 4 + 3 + 8 | 10 | Fillers |  |
| 4 + 5 + 3 | 16 | Fillers |  |
| 4 + 5 + 7 | 12 | Fillers |  |
| 5 + 2 + 3 | 16 | Fillers |  |
| 5 + 2 + 4 | 16 | Fillers |  |
| 5 + 2 + 9 | 13 | Fillers |  |
| 5 + 3 + 2 | 14 | Fillers |  |
| 5 + 3 + 6 | 11 | Fillers |  |
| 5 + 4 + 3 | 16 | Fillers |  |
| 5 + 4 + 7 | 12 | Fillers |  |
| 5 + 9 + 2 | 11 | Fillers |  |
| 6 + 2 + 3 | 15 | Fillers |  |
| 6 + 2 + 7 | 11 | Fillers |  |
| 6 + 3 + 2 | 15 | Fillers |  |
| 6 + 7 + 2 | 9 | Fillers |  |
| 7 + 6 + 2 | 9 | Fillers |  |
| 9 + 5 + 2 | 11 | Fillers |  |
